# Identification and Characterization of Specific Nanobodies against Trop-2 for Tumor Targeting

**DOI:** 10.3390/ijms23147942

**Published:** 2022-07-19

**Authors:** Yaozhong Hu, Yi Wang, Jing Lin, Sihao Wu, Huan Lv, Xuemeng Ji, Shuo Wang

**Affiliations:** Research Institute of Public Health, School of Medicine, Nankai University, Tianjin 300071, China; yzhu@nankai.edu.cn (Y.H.); 2120201268@mail.nankai.edu.cn (Y.W.); 2120201263@mail.nankai.edu.cn (J.L.); 1120210672@mail.nankai.edu.cn (S.W.); lvhuan@nankai.edu.cn (H.L.); jixuemeng@nankai.edu.cn (X.J.)

**Keywords:** Trop-2, nanobody, tumor targeting, proliferation, migration

## Abstract

Trophoblast cell-surface antigen 2 (Trop-2) is a tumor-associated antigen that is connected with the development of various tumors and has been identified as a promising target for tumor immunotherapy. To date, the immunotherapy against Trop-2 mainly relies on the specific targeting by monoclonal antibody in antibody-drug conjugate (ADC). Alternatively, the single domain antibodies of nanobodies (Nbs) possesses unique properties such as smaller size, better tissue penetration, etc., to make them good candidates for tumor targeting. Thus, it was proposed to develop anti-Trop-2 Nbs for tumor targeting in this study. Generally, three consecutive rounds of bio-panning were performed against immobilized recombinant Trop-2, and yielded three Nbs (Nb60, Nb65, and Nb108). The affinity of selected Nbs was determined in the nanomolar range, especially the good properties of Nb60 were verified as a promising candidate for tumor labeling. The binding to native Trop-2 was confirmed by flow cytometry against tumor cells. The inhibitory effects of the selected Nbs on tumor cell proliferation and migration were confirmed by wound healing and Transwell assay. The clear localization of the selected Nbs on the surface of tumor cells verified the potent labeling efficiency. In conclusion, this study provided several Nbs with the potential to be developed as targeting moiety of drug conjugates.

## 1. Introduction

Regular therapeutics of tumor greatly rely on the chemo- or radio-therapy with the consideration of surgical operation. The rapid development of novel targeted therapeutic strategies depends on the identification of targeting moieties of most employed conventional monoclonal antibodies (mAbs) against biomarkers that are highly expressed on tumor cells, rather than normal cells, with the functionalities of promoted tumor growth, proliferation, or metastasis [1]. Varied strategies can be applied to either target the tumor marker directly with antibodies to inhibit the mediated biological effects on tumor cells or develop antibody-dependent conjugate (ADC) to deliver cytotoxic reagents to tumor sites specifically [2]. Thus, different tumor markers will be focused on, based on their bioactivities, as well as the diagnostic and therapeutic strategies.

As one of the tumor markers that has attracted tremendous focus for ADC drug development, Trop-2, also known as tumor-associated calcium signal transducer 2 (TACSTD2), is a cell surface glycoprotein that is highly expressed in malignant tumors such as breast cancer, colon cancer, pancreatic cancer, and gastric cancer with a regulatory role in tumor cell self-renewal, proliferation, and transformation [3,4]. The high level of Trop-2 is well accepted to connect with the poor prognosis of tumor patients [5,6]. The detailed mechanism that are related to Trop-2 mediated tumor growth, proliferation, and metastasis has been unveiled with the recruitment of multiple signaling pathways. The phosphorylation of serine residue (S303) by protein kinase C (PKC) can induce the release of phosphatidylinositol (4,5) bisphosphate (PIP2) and Ca^2+^ from the endoplasmic reticulum to further stimulate mitogen-activated protein kinase (MAPK) signaling and cell cycle progression [7,8]. The interaction of Trop-2 with the potential ligand of Insulin-like growth factor 1 (IGF-1) will prevent IGF-1R signaling [9,10]. The translocation of intracellular domain of Trop-2 after cleavage, and colocalization with β-catenin in the nucleus will upregulate cyclin D1 and cellular-myelocytomatosis viral oncogene (c-myc) to promote tumor growth [11]. The high level of Trop-2 potentially contributes to extracellular regulated protein kinase (ERK) 1/2 phosphorylation, followed by the upregulation of the downstream transcription factor apoptotic protease activating factor-1 (AP-1), which is a target of many tumor-associated genes, such as apoptosis- (B-cell lymphoma-2 (BCL-2), FasL), invasion-, and metastasis-related (matrix metalloproteinases (MMPs), Bordopram, Ezrin) genes. All of the functional mechanisms of Trop-2 on tumor progression make it an attractive biomarker to develop targeting modules for diagnosis or tumor therapy [9,12].

Up to date, therapeutic strategies against Trop-2 have been extensively explored to either inhibit Trop-2 mediated tumor invasion or metastasis with solely employed anti-Trop-2 antibodies or facilitate drug delivery to tumor sites specifically with ADC formats, and most of the pre-clinical or clinical investigation focus on the development of ADC against Trop-2. Mao et al. [13] conjugated a human anti-Trop-2 Fab engineered antibody with doxorubicin (DOX) to form the complex of Trop-2 Fab-DOX to target Trop-2-expressing pancreatic cancer cells with a controlled release of DOX, and the results demonstrated the inhibitory effects on PC cell proliferation and migration with potent antitumor activity. Cardillo et al. [14] conjugated a humanized anti-Trop-2 antibody (hRS7) with SN-38 to active metabolite of irinotecan, Sacituzumab govitecan (IMMU132), which resulted in a novel ADC that can be used for against a wide range of solid tumors, including pancreatic, gastric, triple-negative breast cancer (TNBC), and small-cell and non-small-cell lung carcinomas. The results of IMMU-132 in Phase I/II clinical trials were encouraging, with a clinical benefit rate of 46% in patients with metastatic TNBC; patients experienced Grade 3 or higher adverse events, including anemia (14%), diarrhea (13%), leukopenia (16%), and neutropenia (39%) [15]. It was also well tolerated in epithelial cancer, with Grade 3 toxicities (low back pain and bacteremia) occurring in two of six patients, but no Grade 4 non-hematologic toxicities were observed [16]. The ADC formats that are listed above wholly rely on the preparation of Trop-2-specific mAbs, however, with significant drawbacks that originated from the intrinsic properties of mAbs including the limited tumor penetration and the high cost for production and application, as well as the notable immunogenicity or therapeutic resistance. Alternative candidates of antibody fragments with smaller size and robust characteristics are explored to increase tissue penetration and targeting efficiency, or simply the antibodies with more applicability for ADC development are expected.

The domain antibody fragments of nanobodies (Nbs) are engineered from heavy chain only antibodies (HCAbs) that exist in the peripheral blood of camelid, and are naturally devoid of light chain and the first constant region of heavy chain (CH1). Cloning of the variable domain of the heavy chain (VHH) can produce the antigen- binding reservoir, referred as Nbs with the molecular size of around 15 kDa [17,18]. Unique characteristics of Nbs have been verified by comparing with conventional mAbs. The longer CDR3 regions of Nbs can form the more flexible paratopes that target the unique epitopes on the cognate antigens, which are generally inaccessible for conventional antibodies [19,20]. Moreover, the robust properties of Nbs such as high solubility and inherent domain stability under extreme conditions can ensure the binding activity in the tumor microenvironment [21,22]. The smaller size of Nbs can ensure deep tissue penetration and is considered to be the cornerstone of chimeric antigen and targeted drug delivery. Moreover, the easy manipulation of Nb-drug conjugates can ensure the scaled production and reproducibility for application. In general, the unique properties of Nbs facilitate employment as promising candidates for targeted drug delivery and tumor targeting [23,24].

**Figure 1:**
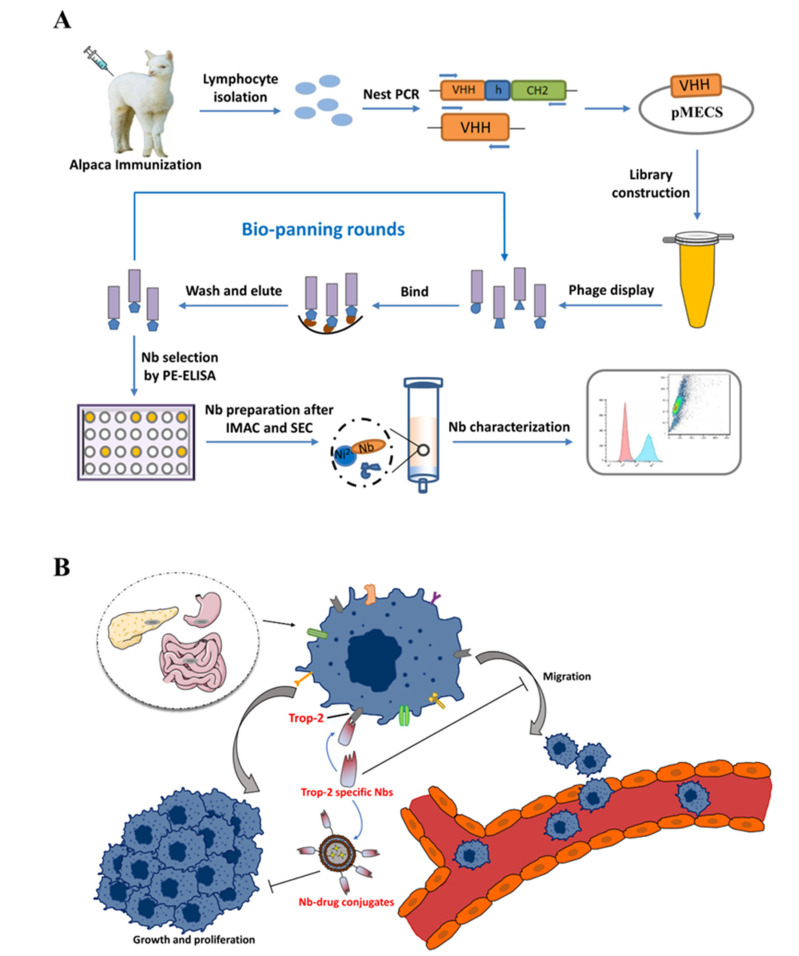
Schematic illustration of Trop-2-specific Nb preparation and verification. (**A**) The general strategy for Nb immune library construction and anti-Trop-2 Nbs selection. (**B**) The general strategy for tumor targeting and inhibition with Trop-2 specific Nbs.

Herein, this study is proposed to develop specific Nbs against Trop-2 and to evaluate the targeting properties of the prepared binders to tumor cells. After the phage displayed selection against recombinant Trop-2 protein, several Nbs were obtained with specific targeting to recombinant Trop-2. The binding to native Trop-2 was then confirmed by labeling tumor cells with selected Nbs. The characteristics of Nbs were evaluated by determining the stability and affinity to prove the robust properties. Finally, the successful localization of Nbs on the surface of the tumor cells has been verified by fluorescence-based imaging. In general, Trop-2 specific Nbs have been identified and evaluated with the potential to serve as a binding moiety for the development of Trop-2-targeting ADC (Figure 1).

## 2. Results

### 2.1. Construction of Nb Library, Panning and Screening

After consecutive immunization with recombinant Trop-2 antigen, an immune Nb library was constructed after cloning VHH encoding genes into pMECS phage-display plasmids, and transformation into *Escherichia coli* (*E. coli*) TG1 cells. The quality of the Nb library was assessed through determining the diversity of the correct percentage of VHH insertion. A diversity of 1.7 × 10^7^ *cfu*/mL was calculated through counting the single colonies of a serial dilution of the library. The percentage of correct insertion was determined by performing colony PCR against 48 randomly selected single colonies, and the bands corresponding to the size of around 600 bp were recognized as the fragments that were amplified from VHH, which revealed the percentage of 95% for VHH insertion (Figure S1). In general, an Nb library was constructed with accepted diversity and a high level of VHH repertoire.

There were three rounds of bio-panning that were performed to enrich Trop-2-specific Nb-displayed phages, and the relative enrichment was confirmed to increase from 1.5 to 12.3 during the consecutive panning rounds, which indicates a good enrichment for target-specific phage particles (Figure S2A).

For the screening of Trop-2-specific Nbs, periplasmic extract-based ELISA (PE-ELIS) was performed against 190 randomly selected single colonies (48 from the first panning) round, 94 from the second round, and 48 from the third round). As shown in Supplementary Materials Figure S2B, 16 binders with potential positive response were confirmed, and divided into three different families according to the amino acid sequence of the post-translational CDR3 region after DNA sequencing, and named Nb60, Nb65, and Nb108 as the representatives of the corresponding families (Figure S2C). Analysis of the amino acid sequence of the selected Nbs revealed their origination from either VH of conventional antibodies or VHH of heavy-chain-only antibodies. The presence of Phe at position 37, Glu at position 44, and Arg at position 45 indicate that the V-domain originated from a heavy-chain-only antibody [25].

### 2.2. Expression and Purification of Selected Nbs

**Figure 2:**
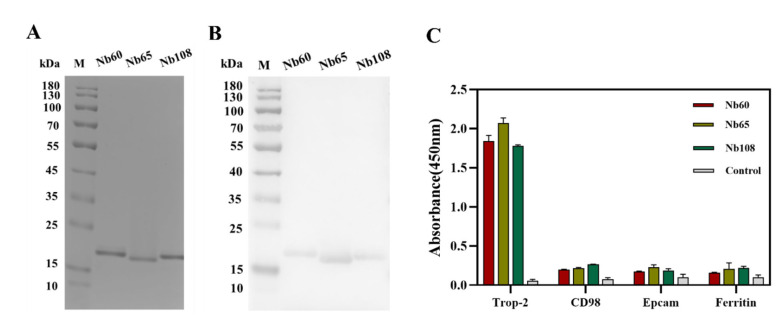
Purification and characterization of the selected Nbs. (**A**) The purity and molecular size of the selected Nbs were verified by SDS-PAGE. (**B**) Western blot analysis was used to confirm the His-tagged Nbs after expression and purification by IMAC and SEC. (**C**) Indirect ELISA was performed to confirm the binding ability and cross-reactivity of the selected Nbs with irrelevant targets (CD98, EpCAM, and Ferritin). All the data are presented as the mean ± standard deviation (n = 3). Each experiment was repeated at least three times.

For the expression and purification of Trop-2 specific Nbs, pMECS plasmids containing the encoding genes were extracted and transformed into *E. coli* WK6 cells to produce Nbs that were fused with Hemagglutinin- (HA-) and His-tag in periplasm with the induction of Isopropyl beta-D-thiogalactopyranoside (IPTG). After the release of PE by osmic shock, the Nbs were purified by immobilized metal affinity chromatography (IMAC) and size exclusion chromatography (SEC). The SEC spectrum revealed a single symmetrical peak, indicating a good purity and homogeneity of Nbs after the two-step purification. The band distribution was further confirmed by sodium dodecyl sulfate polyacrylamide gel electrophoresis (SDS-PAGE), and the results demonstrated the single bands corresponding to the size of around 15 kDa, as expected for the position of the Nbs (Figure 2A). The identity of the Nbs was confirmed by Western blot to verify the presence of His-tag-conjugated Nbs at the expected position (Figure 2B).

#### 2.2.1. Specificity and Potential Cross-Reactivity of Selected Nbs

After preparation of anti-Trop-2 Nbs, the binding specificity and potential cross-reactivity were determined by ELISA against recombinant Trop-2, or other irrelevant targets including CD98, EpCAM, and Serum ferritin. The results demonstrated the significant response from the groups of Trop-2, which indicated the binding of Nbs to the coated antigen, whereas no significant response was observed for the groups of irrelevant proteins to verify the high specificity and low cross-reactivity of the selected Nbs. The results confirmed the targeting of the selected Nbs to Trop-2 specifically (Figure 2C), and indicated these Nbs can be employed as candidates for the development of targeting drug moiety.

#### 2.2.2. Thermal Stability of Nbs

The thermal stability of Nbs is an important property that is closely related to its in vivo applicability and considered as the significant indicator of a good pharmaceutical candidate. To evaluate the thermal stability, melting temperature (Tm) values were determined by performing a Thermofluor assay. The results revealed the data from 67.26 to 70.71 °C (Table S1 and Figure S3) and demonstrated the robust properties of the selected Nbs for endurance of conditions for antibody drug conjugation, and direct in vivo application.

#### 2.2.3. Affinity of Nbs

The binding kinetics of Nbs was analyzed by surface plasmon resonance (SPR) on immobilized recombinant Trop-2 protein. The condition with the best immobilization was investigated to indicate the coating buffer of sodium acetate buffer (pH 4.0). This pH can maximize the degree of immobilization of the antigen and strengthen the electrostatic interaction between the activated ester groups on the sensor surface and the antigen [26]. Then, the binding kinetic test was carried out and the parameters were determined after kinetic fitting based on a 1:1 binding model. As shown in Table S1 and Figure S4, the calculated association constant (ka) for the selected Nbs was between 2.8 × 10^4^ and 1.513 × 10^5^ M^−1^∙s^−1^, and the dissociation constant (kd) ranged from 5.701 × 10^−5^ to 5.355 × 10^−3^ s^−1^. The calculated equilibrium dissociation constants (K_D_) from the ratio of kd/ka were determined to be between 0.7443 and 119 nM. A good property of dissociation is considered as an important characteristic for the application of Nb as the targeting moiety of a drug delivery system, as the binding to the target will be preferred if no or less dissociation occurred. The kinetic parameters revealed Nb60 as a promising candidate as almost no dissociation will happen after binding to the target of the tumor marker.

#### 2.2.4. Binding of Nbs to Native Trop-2

**Figure 3:**
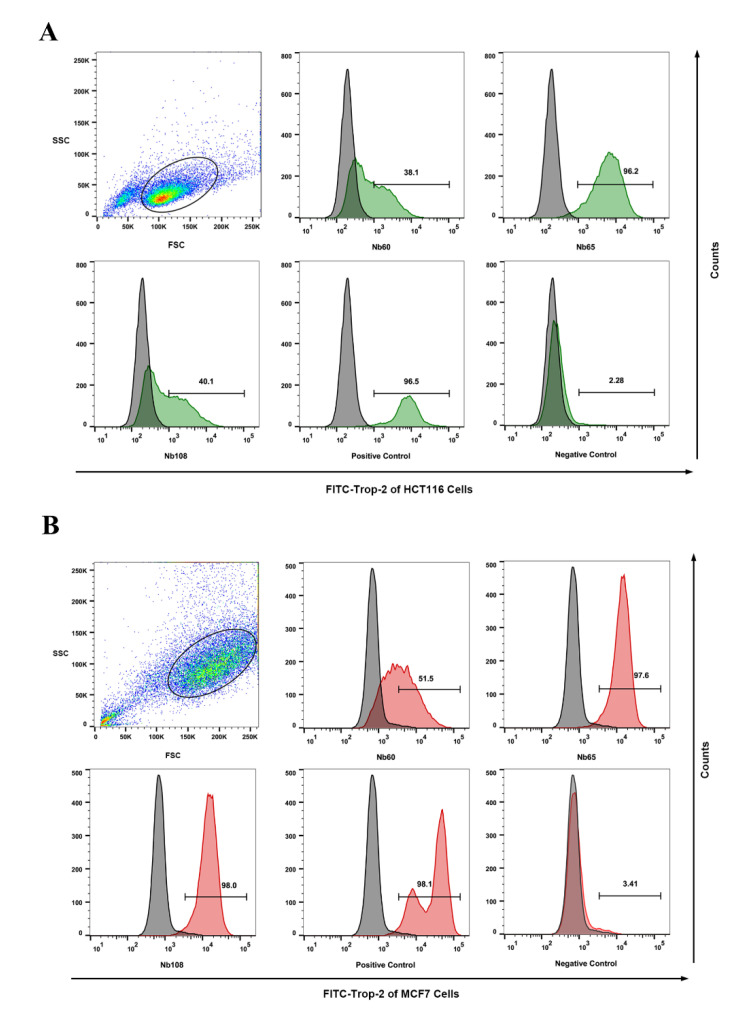
Binding confirmation of the selected Nbs to native Trop-2 by flow cytometric analysis. Histogram plot shift of the selected Nbs was determined in flow cytometry analysis by comparing the staining of Nbs with the groups of positive (anti-Trop-2 IgG) and negative (an irrelevant Nb targeting to β-lactoglobulin). The clear binding of the selected Nbs was confirmed by performing flow cytometric analysis against the tumor cells of HCT116 (**A**) and MCF7 (**B**).

The binding ability of the selected Nbs to native Trop-2 that was expressed on the cells was assessed by flow cytometry against colon rectal cancer cells line of HCT116 and breast cancer cell line of MCF7. The clear shift of the cells that were incubated with Nbs was determined by comparing with the groups of either a blank control that was incubated without Nbs, or negative control that was incubated with an irrelevant Nb. The cells that were incubated with anti-Trop-2 IgG serve as the positive control to determine the gating strategy. The results revealed the clear shift of both tumor cells of HCT116 and MCF7 after incubation with the selected Nbs, which demonstrated the binding of Nbs (Nb60, Nb65, and Nb108) to native Trop-2 that was expressed on the membrane of tumor cells (Figure 3).

**Figure 4:**
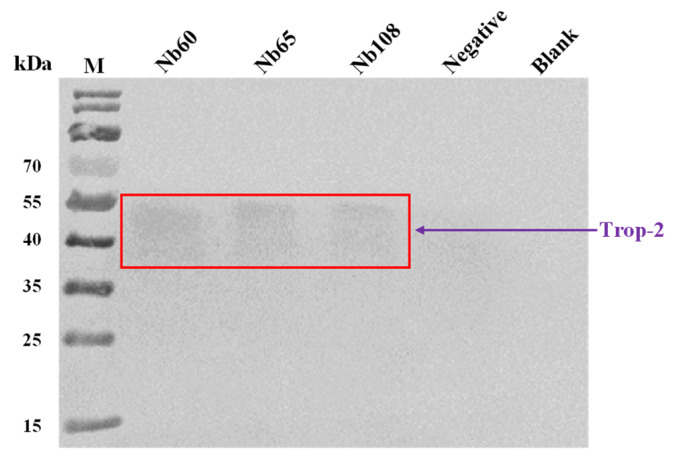
Immunoprecipitation of Trop-2 by Nbs. HCT116 cell lysates were collected and incubated with Nbs for capturing of Trop-2 protein. The protein A/G-coated agarose beads that were conjugated with anti-HA-tag IgG were used to precipitate the complex of Nb and Trop-2. The pellets were subjected to separation by SDS-PAGE and analysis by Western blot. Native Trop-2 was detected with mouse anti-Trop-2 IgG and HRP-conjugated anti-mouse IgG. Immunoprecipitation with mouse anti-HA-tag IgG instead of Nb was used as a negative control. Immunoprecipitation without any antibody was used as a blank control.

The binding of the selected Nbs to native Trop-2 was further confirmed by performing immunoprecipitation. The incubation of Nbs with cell lysate of HCT116 allowed the complex of Nbs with Trop-2. Protein complexes were separated by SDS-PAGE and transferred to nitrocellulose membranes for Western blot analysis. A band corresponding to Trop-2 was seen after incubation with rabbit anti-Trop-2 IgG and HRP-conjugated goat anti-rabbit IgG. The results demonstrated the recognition of native Trop-2 by the selected Nb (Figure 4).

### 2.3. Cytotoxicity and Apoptosis Assays

In order to explore the effect of anti-Trop-2 Nbs on the proliferation of cancer cells, different concentrations of Nbs were incubated with HCT116 cells for a Cell Counting Kit-8(CCK-8) assay to evaluate the cell viability. The results demonstrated the relatively similar signal of anti-Trop-2 Nb-treated cells as the data from the blank or negative controls, which indicated that proliferation of HCT116 cells was not affected by anti-Trop-2 Nbs (Figure S5A).

The apoptosis of HCT116 was analyzed after staining cells with Annexin V and 7-AAD. The cells that were precoated with anti-Trop-2 Nbs were collected and stained with Annexin V and 7-AAD. The cells that were incubated with an irrelevant Nbs targeting to β-lactoglobulin served as the negative control. The cells without incubation with Nbs were used as the blank groups. The apoptosis of HCT116 was determined by comparing the groups of Nbs with a negative and blank control. The results revealed no significant cell apoptosis by comparing with the control group. Thus, it was demonstrated that anti-Trop-2 Nbs were not involved in the apoptosis process of cells (Figure S5B).

### 2.4. Inhibition of Selected Nbs on the Migration of HCT116 Cells

**Figure 5:**
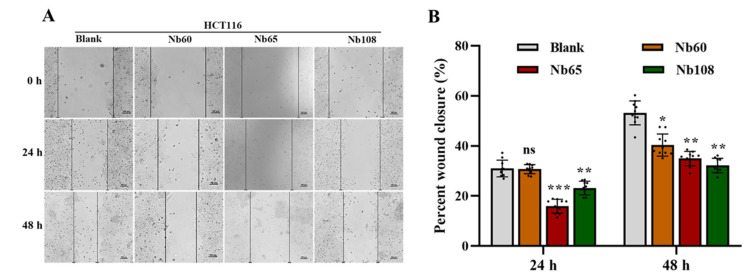
Determination of the inhibitory effect of Nbs in a wound healing assay. (**A**) The wound of the cells was recorded and displayed as indicated (0, 24 h, 48 h). (**B**) The wound healing percentage was indicated in a bar chart according to the wound healing width. The difference between the experimental and blank groups was analyzed using one-way ANOVA followed by Dunnett multiple comparison test. The data are shown as the mean ± SD, and significance was indicated as * *p* < 0.05, ** *p* < 0.01, *** *p* < 0.001.

The migration of tumor cells is the property that significantly contributes to the invasion and metastasis of tumors. In order to determine the inhibitory effect of anti-Trop-2 Nbs on the migration of tumor cells, a wound healing assay was performed to determine the inhibitory efficiency of the selected Nbs on tumor cell migration. As shown in Figure 5, the results of the wound healing experiment revealed the significant inhibitory effect on cell migration upon the presence of anti-Trop-2 Nbs by comparing with the blank group. Treating the cells with Nb60 for 48 h yielded inhibition on cell migration rather than 24 h. The Nb65 treatment could result in the most obvious inhibition of cell migration with the wound healing width of around 13%. After treatment for 48 h, the most significant inhibitory effect of Nb108 was observed with the wound healing width of about 32%. In general, significant inhibition of anti-Trop-2 Nbs on the migration of tumor cells was observed to demonstrate their potential application as theranostic reagents.

**Figure 6:**
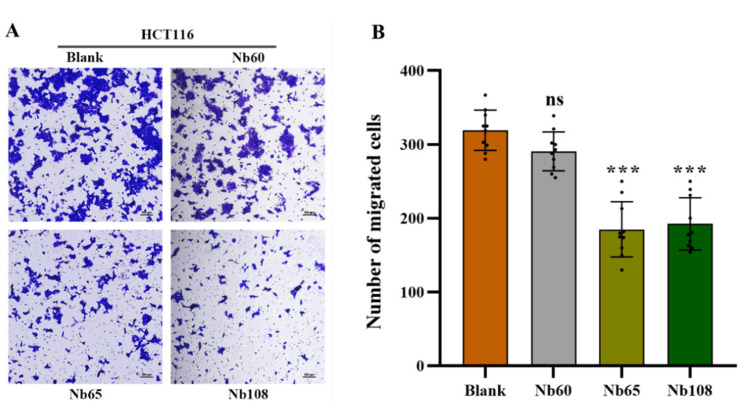
Inhibition of Nbs on cell migration in a Transwell assay. A Transwell assay was performed to examine the potential migration of HCT116 cells, and a clear inhibition can be observed after treating with selected Nbs (**A**). The inhibition of the selected Nbs on cell migration were indicated as a bar chart (**B**). The difference between the experimental and blank groups was analyzed using one-way ANOVA followed by Dunnett multiple comparison test. The data are shown as the mean ± SD, and significance was indicated as *** *p* < 0.001.

The inhibitory effect was further confirmed by a Transwell assay to evaluate the migration ability of HCT116 cells after treatment with anti-Trop-2 Nbs. Upon the incubation of tumor cells with 2 nM Nbs in the upper chamber, the cells that migrated through the membrane were observed to be reduced after determining the signal of violet stained cell clusters. A significant inhibition was observed for the cells that were incubated with Nb65 and Nb108, and the strongest inhibitory could be summarized for Nb65, which is coincident with the result of the wound healing assay. A slight inhibition could be observed for Nb60, whereas this without significant difference (Figure 6).

### 2.5. Localization of Nbs on HCT116 Cells

**Figure 7:**
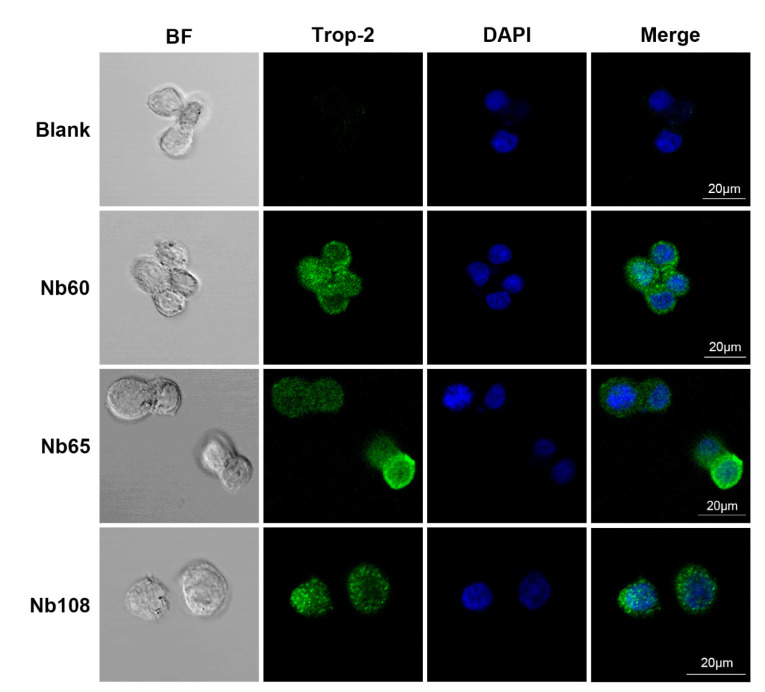
Cell staining of the selected Nbs in confocal immunofluorescence. The HCT116 cells were fixed with 4% paraformaldehyde and blocked with 1% BSA. Then, the Nbs were incubated with cells to allow targeting to the cell surface. Mouse anti-HA monoclonal antibody and Alexa Fluor^®^ 488-conjugated goat anti-mouse IgG were used to stain the displayed Nbs, and DAPI was applied to stain the nucleus.

In order to visualize the binding of anti-Trop-2 Nbs on the membrane of tumor cells, and initially assess their potential as a targeting moiety of drug delivery systems, confocal immunofluorescence was performed against HCT116 cells. The HCT116 cells that were stained with Nbs were fluorescence labelled by incubating with mouse anti-HA IgG and Alexa Fluor^®^ 488-conjugated goat anti-mouse IgG sequentially. After staining of the nucleus with DAPI, the cells were analyzed to visualize either the green fluorescence on the cell surface, or the blue signal of nucleus. The results revealed the appearance of green signals on the surface of the membrane, and merging of the cell images localized the cells and confirmed the successful attachment of Nbs on extracellular domain of Trop-2. The results demonstrated the binding of the selected Nbs on the surface Trop-2 of HCT116 cells (Figure 7), and verified their potential for development as diagnostic or therapeutic agents, as well as the employment as targeting moiety of ADC.

## 3. Discussion

Trop-2 is a transmembrane glycoprotein that is encoded by a tumor-associated calcium signal transducer 2 (TACSTD2) gene that is located on chromosome 1p32. Varied studies have shown the involvement of Trop-2 in multiple growth-stimulating signaling pathways, thereby playing a role in the growth and proliferation of tumor cells. An increased expression level of Trop-2 is closely associated with a poor prognosis of various solid tumors [27,28]. Trop-2 has been selected as the prognostic marker or an attractive target to develop targeting molecules for diagnosis and tumor therapy. To date, the development of anti-Trop-2 theranostic strategies mainly utilize conventional monoclonal antibodies to inhibit tumor growth, or construct ADC to target the tumor cells with specific antibodies and play the inhibitory role through the conjugated chemicals. A mouse monoclonal anti-Trop-2 antibody of RS7-3G11 (RS7) possesses high pancreatic cancer reactivity to human lung squamous cell carcinoma. The humanized format of hRS7 was confirmed with antitumor activity against various tumors in vitro, such as breast cancer, lung adenocarcinoma, ovarian cancer, etc. [29,30,31]. AR47A6.4.2 and Pr1E11 have low internalization activity and high cell surface retention, with robust in vivo antibody-dependent cellular cytotoxicity (ADCC) and/or in pancreatic, colon, breast, and prostate cancer models or complement-dependent cytotoxicity (CDC) activity [32,33]. The Trop-2-targeting ADC drug sacituzumab govitecan that was developed by Immunomedics has shown excellent therapeutic efficacy in a Phase III clinical trial for the treatment of patients with metastatic triple-negative breast cancer (mTNBC) and has been approved by the Food and Drug Administration (FDA) in April 2020 [34]. Such antibody-based strategies benefit from the high specificity and affinity of monoclonal antibodies to target tumor cells, whereas they are often associated with some pharmaceutical, pharmacological, and pharmacokinetic issues such as poor penetration, blood retention, and significant immunogenicity compared with antibody fragments or small molecules [35]. Nanobodies have been verified with excellent tissue penetration, making them ideal tools for tumor-targeted therapy, especially as the targeting modules of a drug delivery system or developed of ADC [36].

Herein, it was proposed to develop anti-Trop-2 Nbs in this study and investigate their properties to evaluate their potential as candidates for Trop-2 targeting and development as theranostic agents initially. Several anti-Trop-2 Nbs have been selected based on phage display technology from a pre-constructed immune Nb library. The robust properties of the selected Nbs have been confirmed to possess high stability and good solubility. The binding of the selected Nbs to native Trop-2 has been verified by performing flow cytometry against various tumor cells or precipitation from cell lysates, which demonstrated their potential to target Trop-2 for in vivo application. The kinetic analysis revealed the high affinity of the selected Nbs to recombinant Trop-2 protein with K_D_ from pM to nM. However, both of the association and dissociation rates need to be investigated for the following research and application. For the development of a targeted drug delivery system, the low rate of dissociation is preferred as the release of Nbs from the surface Trop-2 will not happen or just occur quite slowly, which allows the relatively prolonged release of therapeutic chemicals in tumor sites to have anti-tumor effects. SPR analysis revealed a very slow dissociation rate (5.701 × 10^−5^ M^−1^∙s^−1^) of Nb60, as well as the high association rate, which initially confirmed the potential of Nb60 as the attachment carrier for the development of ADC. Nb65 and Nb108 are promising candidates of diagnostic agents as the binding happened immediately upon interaction and then dissociated from the target in a short while. The inhibitory effect of the selected Nbs on tumor cells were confirmed to effectively inhibit tumor cell migration after targeting of the Nbs to the surface Trop-2. However, no significant effect of Nbs on cell viability or apoptosis was observed, which indicated less contribution of Nbs to cell growth and proliferation after binding to extracellular Trop-2.

## 4. Materials and Methods

### 4.1. Materials, Strains and Vecotrs

Complete Freund’s Adjuvant, Incomplete Freund’s Adjuvant, and Amicon Ultra Centrifugal Filter Units were from Sigma Aldrich (St. Louis, MO, USA). Lymphoprep™ density gradient medium was purchased from STEMCELL Technologies (Vancouver, BC, Canada). 96-Well Cell Culture Plates and 96-well Microplates were purchased from Corning (New York, NY, USA). SYPRO^®^ Orange Protein Gel Stain, HisPur^TM^ Ni-NTA Resin, Mouse anti-HA IgG, and horseradish peroxidase (HRP) conjugated goat anti-mouse IgGs were all purchased from Thermo Fisher Scientific Inc. (Carlsbad, CA, USA). PCR Purification Kit and Gel Extraction Kits were purchased from QIAquick (Hilden, Germany). TIAN prep Mini Plasmid Kit for plasmid preparation was purchased from TIANGEN (Beijing, China). Tween-20 and 3,3,5,5′-Tetramethylbenzidine (TMB) Two-Component Substrate solution were purchased from Solarbio (Beijing, China). Dulbecco’s modified Eagle’s medium (DMEM), fetal bovine serum (FBS), and penicillin-streptomycin (P/S) were purchased from Gibco (Paisley, UK). Unless otherwise stated, all the reagents that were used in this study were of analytical grade. The antigen of human Trop-2 extracellular recombinant protein was purchased from ACRO Biosystems (Beijing, China) with the purity higher than 95%. *E. coli* TG1 competent cells were used for construction of the immune library and purchased from Lucigen (Middleton, WI, USA). *E. coli* WK6 cells were used for Nb expression and from the lab strain collection. pMECS phagemids were used for the preparation of Nb-displayed phage reservoir, and the expression of Nbs fused with HA- and His-tag. The plasmids were stocked in the lab collection.

### 4.2. Cell Culture

The colorectal cancer (CRC) cell line HCT116 and breast cancer cell line MCF7 were stocked in the lab collection. The cells were cultured in DMEM that was supplemented with 10% FBS, 100 μg/mL P/S, and incubated at 37 °C in a humidified environment with 5% CO_2_.

### 4.3. Immunization and Construction of a Nb Library

Immunization and construction of the Nb library were conducted after following a well-established method with certain modifications [37]. In general, 100 μg of Trop-2 was used for each injection, and subcutaneously injected into a young alpaca after complete emulsification with equal volume of complete or incomplete Freund’s adjuvant in a 6-week duration with one injection per week. A total of three days after the last boost, 50 mL of anticoagulated peripheral blood were collected from the jugular vein of alpaca and subjected to the following procedure immediately to extract peripheral blood lymphocytes by using density gradient centrifugation in a SepMate^TM^ centrifuge tube. Then, the total RNA was extracted from the obtained lymphocytes after complete homogenization with TRIzol^TM^ reagent. The extracted RNA was used as the template to prepare first strand cDNA to serve as the template in the following nested PCR. The fragments encoding VH-CH1-CH2, and VHH-CH2 were amplified simultaneously in the first round PCR by using primers of CALL001 and CALL002 to yield the amplicons with the size of around 900 bp and 700 bp, respectively. The bands were then separated by agarose gel electrophoresis to facilitate the extraction of the fragments corresponding to the size of around 700 bp by using QIAquick gel extraction kit, and serve as the template in the second round of PCR to amplify fragments solely encoding VHH with the size of around 400 bp by using primers of PMCF and A6E to introduce restriction enzyme sites of Pst I and Not I. The recombinant plasmids were constructed after incorporating digested VHH fragments into pMECS phagemids that were digested with the same restriction enzymes of Not I and Pst I. The yielded recombinant plasmids were then electro-transformed into *E. coli* TG1 competent cells to construct the immune library with enough Nb repertoire. The diversity of the library was determined by counting the number of colonies that were produced from the gradient diluted transformants, and 48 single colonies were randomly chosen to determine the percentage of VHH insertion by performing colony-PCR with primers of MP57 and GIII. The colonies were collected in LB medium with supplementation of 10% (*v*/*v*) glycerol and stocked at −80 °C until further use.

### 4.4. Bio-Panning and Screening of Specific Nbs

There were three rounds of bio-panning that were performed to enrich phage particles that were displayed with target-specific Nbs according to the well-described approaches with certain modifications [38]. Briefly, a representative aliquot of the constructed immune Nb library was inoculated into 300 mL of 2 × TY medium to allow the growing until exponential phase. Then, 1 mL of M13KO7 helper phages with the concentration of around 1.0 × 10^12^ *pfu*/mL were supplemented to infect TG1 cells during the incubation at 37 °C without shaking. The TG1 cells that were infected by helper phages were further selected by resuspending the pelleted cells in 2 × TY medium that was supplemented with the antibiotics of kanamycin (Kan), and phages that were encapsulated with the recombinant phagemids were extensively released after overnight shaking. The Nb-displayed phage particles that were present in the cultural supernatant were collected after centrifugation and precipitated with PEG/NaCl solution (200 g PEG + 146.1 g Nacl, to bring to 1 L with Milli-Q H_2_O) on ice. The precipitated phages were then pelleted by centrifugation and resuspended in 1 mL sterile PBS. The recombinant Trop-2 protein (10 μg/mL) was pre-coated on microtiter plates overnight at 4 °C, and blocked with 3% skimmed milk. Then, around 1.0 × 10^11^ *pfu* of the obtained phages were added into the wells of positive and negative, respectively, to allow the interaction of the displayed Nbs with the pre-coated antigen protein. The unbound phages were removed after washing with PBST (PBS containing 0.05% Tween-20), and TEA solution (100 mM triethylamine, pH 11.0) (100 μL) was used to elute to the remaining phage particles. The pH shock was neutralized after mixing the elution with 100 μL of Tris-HCl (1.0 M, pH 7.4) immediately. A total of 10 μL of phage elution were subjected to infect 90 μL of TG1 cells for relative enrichment determination. The remaining phages were used to infect the TG1 cells for the amplified preparation of phages that were used in the next round of bio-panning.

After three consecutive rounds of bio-panning, the potential positive Nbs were screened against recombinant Trop-2 protein in a PE-ELISA. Generally, 190 single colonies were randomly picked up from the plates of the panning rounds and inoculated in 100 μL of 2 × TY medium containing 10% (*w*/*v*) glycerol, 2% (*w*/*v*) glucose, and 100 μg/mL ampicillin. After overnight incubation, 10 μL of cultural medium was transferred to the deep-well plates containing 1 mL of 2 × TY medium that was supplemented with 0.1% (*w*/*v*) glucose and 100 μg/mL ampicillin and grown until the exponential phase with shaking. IPTG was added to reach a final concentration of 1 mM to induce the expression of Nbs with His and Hemagglutinin (HA)-tags. Then, the cell lysates containing Nbs were released by freeze-thaw cycles and used as the reservoir of Nbs for antigen binding. The potential binding properties of Nbs were validated after adding mouse anti-His IgG, alkaline phosphatase (AP) conjugated goat anti-mouse IgGs sequentially, and the developing substrate of p-nitrophenyl phosphate disodium to yield the response at 405 nm. The positive colonies were defined as the binders providing an absorbance at least 2 times of the response from the negative controls. The determined positive colonies were subjected to the following plasmid extraction and nucleotide sequencing.

### 4.5. Expression and Purification of Specific Nbs

The vectors containing the encoding genes of positive Nbs were extracted and transformed into *E. coli* WK6-competent cells for periplasmic expression and purification. Generally, the cells were inoculated in 300 mL terrific broth (TB) medium containing 0.1% (*w*/*v*) glucose, 100 μg/mL ampicillin, and 2 mM MgCl_2_, and incubated at 37 °C until the optical density of 0.6–0.9 at 600 nm was reached. IPTG was added to a final concentration of 1 mM to induce the production of Nbs during overnight incubation at 28 °C. The cells were collected by centrifugation on next day, and periplasmic extracts were released by osmotic shock. PE was subjected to a two-step purification approach including the Ni^2+^ based immobilized metal affinity chromatography (IMAC) and size exclusion chromatography (SEC). In the first step of purification, His-tagged Nbs were covalently absorbed by Ni^2+^ that was decorated on agarose resin, and unbound or unspecific fractions were washed off by several washing steps with PBS containing 25 mM imidazole. Then, the bonded Nbs on the resin were eluted with elution buffer (PBS containing 500 mM imidazole, pH 7.4). The Nb fractions were then subjected to a Superdex^TM^ 75 10/300 GL gel filtration column (GE Healthcare, Waukesha, WI, USA) for size-based separation of the Nbs with other contaminant proteins. The fractions corresponding to the size of Nbs were collected and analyzed by performing SDS polyacrylamide gel electrophoresis (SDS-PAGE) and Western blot to determine the purity and identity. The yielded Nbs were then aliquoted and stocked with the concentration of 1 mg/mL at −80 °C for further investigation.

### 4.6. Characterization of Selected Nbs

#### 4.6.1. Binding Capacity and Cross-Reactivity of Nbs

The binding capacity and cross-reactivity of the selected Nbs were verified by enzyme-linked immune sorbent assay (ELISA) against the target of recombinant Trop-2, and other irrelevant proteins including CD98, EpCAM, and serum ferritin. Briefly, the mentioned proteins (1.0 μg/mL) were coated in a 96-well microtiter plate overnight at 4 °C, and the remaining binding sites were blocked with 3% skim milk for 1 h at room temperature on the next day. After the washing steps with PBST, the Nbs (10 μg/mL) were added and incubated at RT for 1 h to allow the binding to the coated antigens. Mouse anti-HA IgG and HRP-conjugated goat anti-mouse IgGs were incubated sequentially and employed as the primary and secondary antibodies, respectively. 3,3′,5,5′-tetramethylbenzidine (TMB) was applied for color development to facilitate the reading of the response signal at 450 nm with a microplate reader (Tecan, Switzerland) after stopping of the reaction with 2 M H_2_SO_4_ solution. The binding properties were evaluated by comparing the absorbance values from the different groups.

#### 4.6.2. Thermostability of Nbs

The thermostability of the Nbs was measured by the Thermoflor in CFX Connect™ Real-Time PCR Detection System (Bio-Rad, Hercules, CA, USA) to reflect the robustness of the selected Nbs. Generally, Nbs were concentrated to a final concentration of 1.5 mg/mL by using an Amicon ultracentrifugal filter unit, and a reaction system was prepared with the fractions including 25 μL of Nb and 5 μL of 100-fold diluted SYPRO^®^Orange Protein Gel Stain (Sigma, St. Louis, MO, USA). Each assay was prepared in triplicates, and the group with PBS and SYPRO^®^ Orange Protein Gel staining was set up as a baseline control. The device was programmed to increase the temperature from 25 to 95 °C at a rate of 0.5 °C/min. The thermostability was determined by calculating the temperature that yielded half of maximum signal or referred as the melting temperature (Tm). The data was analyzed using Graphpad Prism software version 8.00 (GraphPad Software, San Diego, CA, USA), and the Tm was determined after non-linear fitting.

#### 4.6.3. Affinity of Nbs

The affinity of Nbs was determined by SPR using a Biacore^TM^ X100 instrument (GE Healthcare, Freiburg, Germany). The recombinant Trop-2 was coupled to a CM5 sensor chip by using an amine coupling kit. The appropriate pH was determined by performing pH scouting to yield the best response curve. The SPR analysis was performed at 25 °C with the running buffer of HBS (20 mM of HEPES pH 7.4, 150 mM of NaCl, 0.005% Tween-20, and 3.4 mM of EDTA). The Nbs were injected sequentially as the settled order of serial dilutions (2-fold dilution from 250 to 2 nM) at a rate of 10 μL/min. The association and dissociation steps were performed in 300 s and 600 s, respectively. A regeneration step of 35 s at 30 μL/min was set up by using 10 mM Glycine at pH 2.5, and an additional stabilization in 300 s was guaranteed. The affinity was determined after performing kinetic analysis by using a mathematical fitting of a 1:1 binding model using the BIACORE X100 Evaluation software version 2.0.2 (GE Healthcare, Freiburg, Germany), and the ratio of kd/ka was defined as the equilibrium dissociation constant (K_D_).

#### 4.6.4. Binding of Nbs to Native Trop-2

In order to confirm the binding of the selected Nbs to native Trop-2 on tumor cells, flow cytometry analysis was performed to identify the targeting of the obtained Nbs to the surface of the tumor cells. Trop-2 expression on the HCT116 cells and MCF7 was firstly confirmed by flow cytometry by using anti-Trop-2 IgG (Abcam, Cambridge, UK), and Alexa Fluor^®^ 488-conjugated goat anti-rabbit IgG (Thermo, Waltham, MA, USA). For binding analysis of the selected Nbs to HCT116, around 1 × 10^6^ cells in the exponential phase were washed and resuspended in PBS. A total of 1 μg of Nbs or irrelevant antibody (as negative control) was added and incubated for 30 min. After the washing steps, the cells were resuspended for staining with mouse anti-HA IgG and Alexa Fluor^®^ 488-conjugated goat anti-mouse IgG sequentially in 30 min of each incubation. The positive control was set up by staining with anti-Trop-2 IgG and Alexa Fluor^®^ 488 goat anti-rabbit IgG (Abcam, UK). All the steps were performed on ice, and the cells were finally subjected to analysis on a FACS Callibour (BD Biosciences, Franklin Lakes, NJ, USA) instrument, and the data were analyzed by FlowJo software version 10.6.2 (FLOWJO, Ashland, OR, USA).

In order to further confirm the binding of the selected Nbs to native Trop-2, immunocapture was performed to visualize the Nb-mediated precipitation of Trop-2 from the cell lysate of HCT116. In general, the HCT116 cells were grown to near confluence, and then collected and lysed in RIPA lysis buffer with the supplementation of 1% PMSF protein inhibitor. The experiments were set up to include Tube A containing 20 µL of lysate supernatant and 10 µg of Nbs, and Tube B with 2 µL of mouse anti-HA-tag IgG and 10 µL of protein A/G plus agarose beads (Santa Cruz Biotechnology, Dallas, TX, USA) in 200 µL immune precipitate buffer (IPB, 40 mM Tris pH 8.0, 1% Triton, 10% glycerol, 280 mM NaCl) at 4 °C for 1 h. The group with irrelevant Nb or with only the cell lysate served as the negative and blank control, respectively. Then, the antibody-coated protein A/G beads were washed once with IPB buffer, and resuspended in cell lysates with or without Nbs and incubated overnight at 4 °C. On the next day, the bead-antibody-antigen complexes were washed 5 times with IPB buffer and solubilized in 40 μL of loading buffer for separation by SDS-PAGE, and then analyzed by Western blot to visualize the bands corresponding to the size of Trop-2 with rabbit anti-Trop-2 IgG and HRP-conjugated goat anti-rabbit IgG, respectively.

#### 4.6.5. Wound Healing Assay

The cells in logarithmic growth phase were seeded in a 6-well plate and grown until around 100% confluence. The cell layer was scratched to form a wound with a sterile pipette tip. The suspended cells were washed off with PBS and immersed in DMEM with 1% FBS. Varied amounts of Nbs were added to incubate for 48 h, and the wound was recorded by using an inverted fluorescence microscope at different time points including 0 h, 24 h, and 48 h. The cells that were incubated without Nbs served as the blank control. The speed of cell migration and wound closure were quantified by calculating the wound width with Image J software version 1.8.0 (NIH, Bethesda, ML, USA) [39].

#### 4.6.6. Migration Assay

The inhibition of Nbs to cell migration was further investigated by performing a Transwell assay. In brief, 1 × 10^5^ HCT116 cells in DMEM containing 1% FBS were seeded into the upper chamber of Transwell inserts (8-μm pore size; Corning Inc., Corning, NY, USA) with supplementation of 2 mM of Nbs, and the complete culture medium containing 10% FBS was added to the lower chamber as a chemoattractant. After 36 h incubation in a humidified incubator at 37 °C and with 5% CO_2_, the Transwell chambers were taken off to discard the medium and washed with PBS. Then, the cells were fixed in 4% paraformaldehyde for 15 min at room temperature (RT), and stained with 0.1% crystal violet for 20 min. The upper unmigrated cells were gently wiped off with a cotton swab and the remaining cells were visualized and recorded with an inverted fluorescence microscope. Meanwhile, the stained cells in randomly selected visual fields were recorded for analysis of cell migration.

#### 4.6.7. Cell Viability Assay

The toxicity of Nbs to HCT116 cells was assessed by performing a cell viability assay with cell counting kit-8 (CCK-8). Generally, HCT116 cells in the exponential growth phase were seeded in 96-well plates (cells/mL) and incubated overnight with supplementation of different concentration of Nbs. The groups without supplementation of Nbs was set up as the blank control, and an irrelevant Nbs was used in a negative control. After 48 h treatment, 10 μL of CCK-8 solution was added to each well and then incubated for 2 h at 37 °C. The OD value was measured at 450 nm by using a microplate reader.

#### 4.6.8. Apoptosis Assay

The HCT116 cells in the exponential growth phase were seeded in 6-well culture plates until adherence. After washing with PBS, serum-free DMEM containing 2 nM of Nbs were added for incubation of 48 h. The cells that were incubated with an irrelevant Nb targeting to β-lactoglobulin served as the negative control, and the cells without treating with Nbs were used as the blank control. The cells were collected in flow tubes to incubate with Annexin V/PE at RT for 15 min, and 7-AAD was used to stain mechanistically-induced dead cells before measurement on the flow cytometer (BD Biosciences, Franklin Lakes, NJ, USA). The data were analyzed using FlowJo software version 10.6.2 (FLOWJO, Ashland, OR, USA).

#### 4.6.9. Cell Staining of Selected Nbs

In order to visualize the labeling of the selected Nbs on the surface of HCT116 and verify the potential application as a targeted moiety in drug delivery systems, confocal immunofluorescence was performed. In general, the HCT116 cells were cultured on tissue-treated cell slides at 37 °C to until complete adherence. The cells were then fixed with 4% paraformaldehyde for 10 min at RT, and blocked with 1% BSA for 1 h. Then, 1 μg of Nbs were added and incubated overnight at 4 °C. After washing with sterile PBS, the cells were incubated with mouse anti-HA monoclonal antibody (1:500 dilution) and Alexa Fluor^®^ 488-conjugated goat anti-mouse IgG (1:500 dilution) sequentially, and DAPI was applied to stain the nucleus. Fluorescence images were captured using a laser scanning confocal microscope (LSM880 with Airyscan, Germany) for FITC, and the images of the surface staining were merged into nucleus-stained graphs to localize the binding of Nbs to the surface of the tumor cells.

### 4.7. Statistical Analysis

Statistics were acquired by GraphPad Prism 8 software. The data and error bars represent the mean ± SD that was calculated from the results from the different groups. A significant difference was indicated by one-way ANOVA followed by Dunnett multiple comparison test, and values of *p* < 0.05 were deemed to be a statistically significant.

## 5. Conclusions

We have successfully developed several Nbs against Trop-2. The robust properties have been characterized including the high stability and affinity, with relatively high yield in prokaryotic expression platform. The binding of the selected Nbs to native Trop-2 that was expressed on the surface of tumor cells has been verified. The therapeutic effect of the selected Nbs has been investigated to inhibit tumor cell migration significantly, although no effect on cell viability or apoptosis was observed. The localization of Nbs on the surface of tumor cells after confocal immunofluorescence initially confirmed their potential as a targeting moiety of drug delivery systems. In terms of diagnostic applications, Nb-labeled dyes or conjugated with green fluorescent protein have great potential as ideal tracers for non-invasive in vivo imaging of tumors and lesions. Further investigation of in vivo targeting of sole Nbs or Nb-drug conjugates has been proposed to validate the above potential applications of the obtained Nbs in this study. In terms of therapy, Nb-loaded drugs will enable targeted cancer therapy. The following has been proposed to evaluate the inhibitory effects of these Nbs on tumor growth and metastasis by in vivo analysis, as well as the potential as a targeting moiety of antibody drug conjugates. The research is ongoing and in vivo analysis is in progress to evaluate the targeting of these Nbs in tumor-bearing nude mice, and the inhibitory efficiency.

## Supplementary Materials

The following supporting information can be downloaded at: https://www.mdpi.com/article/10.3390/ijms23147942/s1.
