# Supplementary material for: Identification and Characterization of Specific Nanobodies against Trop-2 for Tumor Targeting
Source: Int J Mol Sci. 2022 Jul 19;23(14):7942. doi: 10.3390/ijms23147942 (PMC9316174; doi:10.3390/ijms23147942)
Supplement: Supplementary file 1 [file ijms-23-07942-s001.zip › ijms-1812682-supplementary.pdf]

Supporting Information

Identification and Characterization of Specific Nanobodies against Trop-2 for Tumor Targeting

Yaozhong Hu[1,#], Yi Wang[1,#], Jing Lin[1], Sihao Wu[1], Huan Lv[1], Xuemeng Ji[1], Shuo Wang[1,*]

1. Research Institute of Public Health, School of Medicine, Nankai University, Tianjin 300071, China.

[#] These authors (Yaozhong Hu and Yi Wang) contribute equally to this work.

[*] Corresponding author,

wangshuo@nankai.edu.cn

Tel.: +86 22 85358445

Table S1 Properties of selected Nbs

Nbs	MW/	pI	Tm value [a]	k_a	k_d	K_D [b]
	kDa	-	℃	$M^{-1}\cdot s^{-1}$	s^{-1}	nM
**Nb60**	16.782	6.30	70.71 ± 0.08	7.660×10^4	5.701×10^{-5}	0.7443
**Nb65**	16.275	6.64	60.97 ± 0.12	1.513×10^5	5.355×10^{-3}	35.39
**Nb108**	15.456	6.30	67.26 ± 0.18	2.800×10^4	3.327×10^{-3}	118.8

[a]: Thermal stability of selected Nbs were represented as Tm value generated from 3 repeated assay, and results are represented as mean ± SD (n = 3).

[b]: The affinity of the selected Nbs was expressed with K_D, the K_D from the ratio of k_d/k_a.

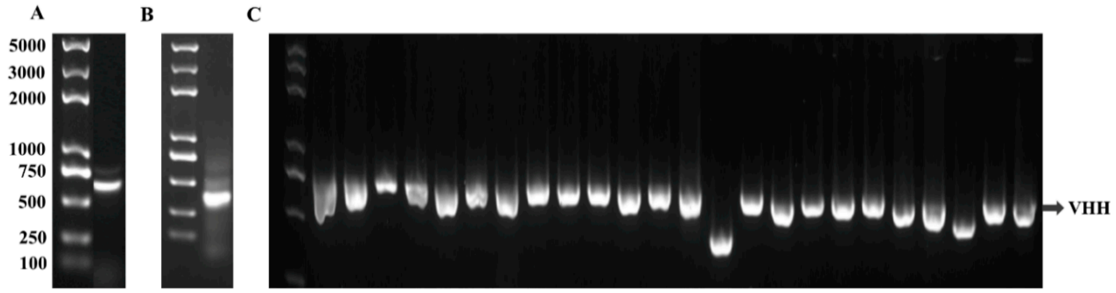

Figure S1. Construction of the immune Nb library.

(A) Fragments amplified after 1st PCR. Two bands were visualized with the size of around 900 and 700 bp, respectively. (B) VHH fragments amplified after 2nd PCR with the size of around 400 bp. (C) The percentage of VHH insertion was determined by colony PCR.

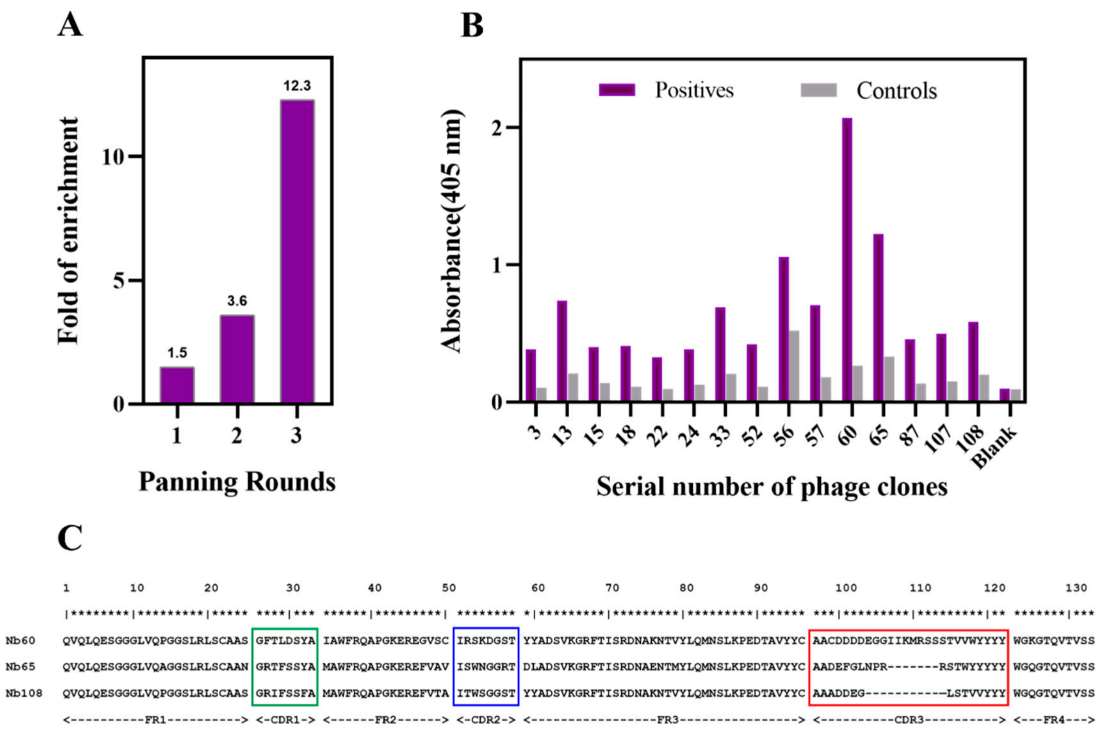

Figure S2. Specific Nbs retrieved from the immune libraries.

(A) Relative enrichment of Trop-2-specific Nbs was determined after each round of bio-panning by comparing negative control. (B) Positive colonies identified from PE-ELISA with signal at least two-fold higher than the corresponding blank control. (C) Three Trop-2-specific Nbs with distinct amino acid sequences were identified. Alignment of the amino acid sequences of Nbs was indicated as the complementarity determining regions (CDRs) and framework regions (FRs).

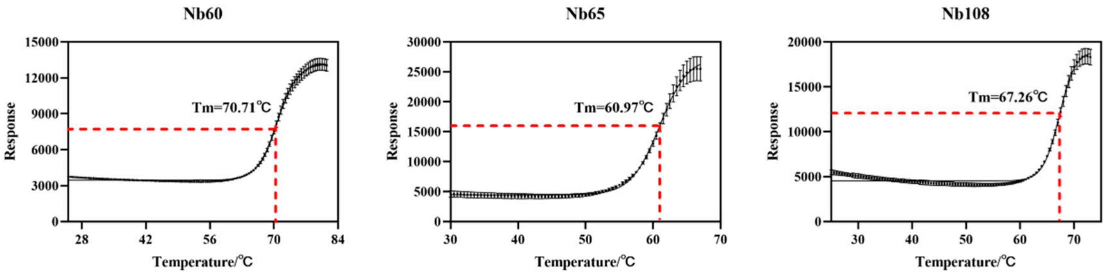

Figure S3. Thermal stability of selected Nbs.

All data plotted are expressed as mean ± SD (n = 3). Repeated at least 3 times for every test.

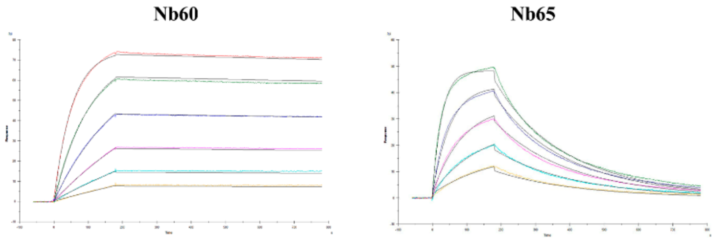

Figure S4. Binding affinity of selected Nbs.

The affinity of selected Nbs were determined by performing SPR analysis with the running buffer of HBS. The Nbs were injected sequentially as the settled order of serial dilutions (2-fold dilution from 250 to 3.91 nM). The kinetic analysis was analyzed by fitting on a 1:1 binding model, and the association and dissociation rate were determined, respectively.

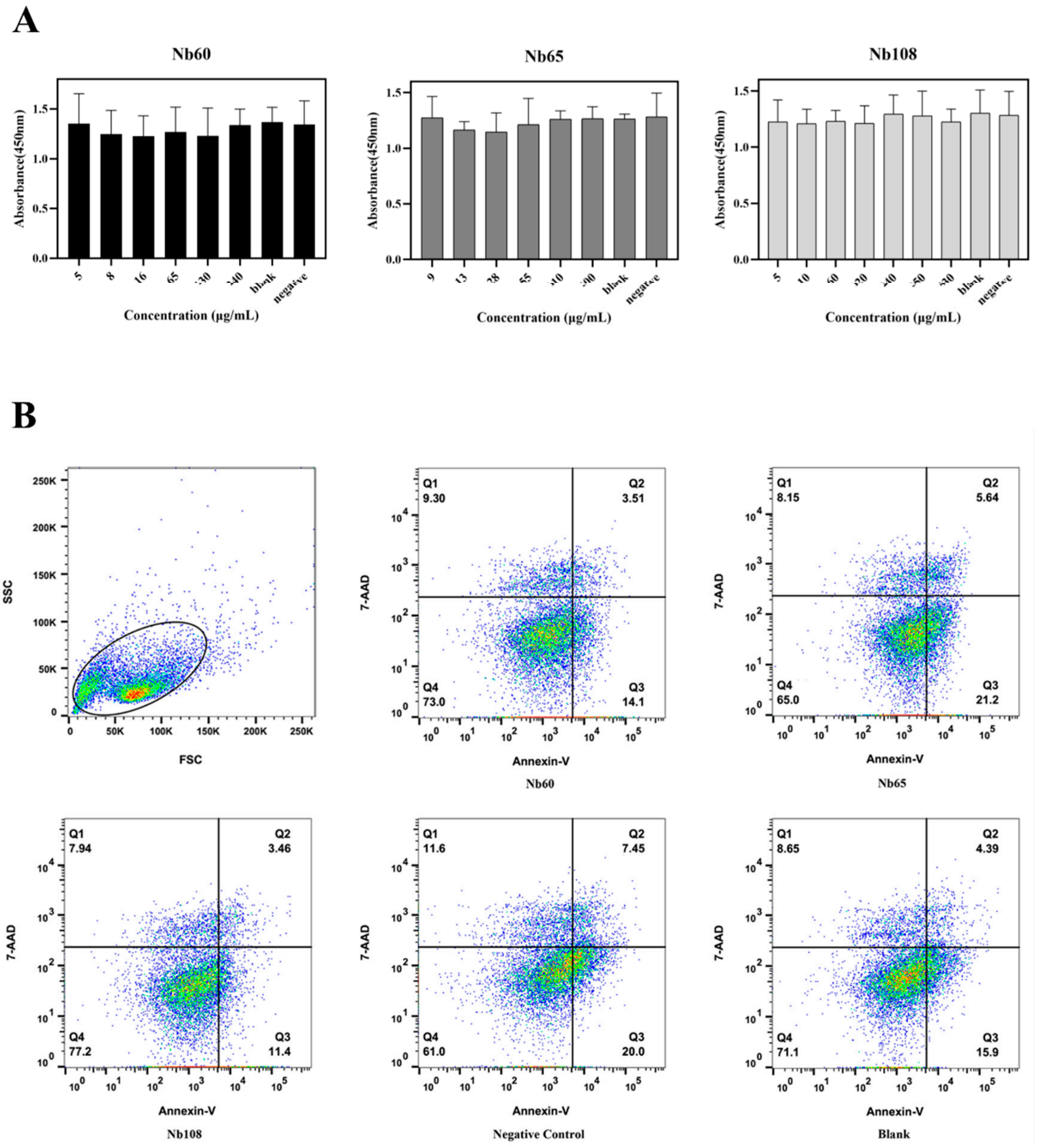

Figure S5. Cytotoxicity and apoptosis assays

(A) The viability of HCT116 cells after treating with anti-Trop-2 Nbs was detected by CCK-8 method. (B) Cell apoptosis was determined by flow cytometric analysis by staining Nb treated cells with Annexin V and 7-AAD
